# Effects of astragalus injection on the TGFβ/Smad pathway in the kidney in type 2 diabetic mice

**DOI:** 10.1186/1472-6882-14-148

**Published:** 2014-05-05

**Authors:** Yanna Nie, Shuyu Li, Yuee Yi, Weilian Su, Xinlou Chai, Dexian Jia, Qian Wang

**Affiliations:** 1 School of Preclinical Medicine, Beijing University of Chinese Medicine, 100029 Beijing, China

## Abstract

**Background:**

In traditional Chinese medicine, astragalus injection is used to treat diabetic nephropathy (DN). The current study was conducted to determine the effects of astragalus injection on DN by assessing potential modulation of the transforming growth factor beta TGFβ/Smad signaling pathway.

**Methods:**

Diabetic, male KKAy mice, aged 14 weeks were randomly divided into a model group and an astragalus treatment group, while age-matched male C57BL/6J mice were selected as controls. The treatment group received daily intraperitoneal injections of astragalus (0.03 ml/10 g.d), while the model group received injections of an equivalent volume of saline. Mice were euthanized after 24 weeks. Serum samples were obtained from animals in each group, and blood glucose, creatinine, and urea nitrogen levels were measured. Tissue samples from the kidney were used for morphometric studies. The expression of TGFβ1, TGFβR-Ι, Smad3, and Smad7 were evaluated using reverse transcription-polymerase chain reaction (RT-PCR), and western blot analysis.

**Results:**

Mice in the model group became obese, and suffered complications, including hyperglycemia, polyuria, and proteinuria. Astragalus treatment significantly reduced albuminuria, improved renal function, and ameliorated changes in renal histopathology. Moreover, administration of astragalus injection increased Smad7 expression, and inhibited the expression of TGFβR-Ι, Smad3 and its phosphorylation, and decreased the mRNA level of TGFβ1.

**Conclusions:**

The TGFβ/Smad signaling pathway plays an important role in the development of DN. Administration of astragalus injection could prevent or mitigate DN by rebalancing TGFβ/Smad signaling, and could play a protective role in DN-induced renal damage in KKAy mice.

## Background

Diabetic nephropathy (DN) is a major microvascular complication of diabetes mellitus, as well as the leading cause of end-stage renal disease [1]. A pathological change associated with DN is the accumulation of normal and abnormal extracellular matrix components (ECM) in renal glomeruli and interstitium [2]. TGFβ is a secreted protein that plays a critical role in renal fibrosis and the accumulation of ECM [3], and intraperitoneal injections of TGFβ alone were sufficient to initiate a prominent renal fibrotic response [4]. Further, TGFβ isoforms and their receptors were upregulated in both experimental models of DN, as well as in human DN [5,6]. We chose to focus on TGFβ1 because it is the most abundantly expressed isoform in the kidney, and has been most closely linked to the pathophysiology of DN [6]. Additionally, it was reported that TGFβ1 was stimulated by high glucose levels, and was chiefly expressed in renal tubular epithelial cells in diabetic mice [7].

TGFβ1 binds to the TGFβR-IΙ, which in turn recruits the binding of TGFβR-I to form a heterotetramer. TGFβR-I subsequently phosphorylates the Smad proteins [8,9], after which the activated Smad2/Smad3 associates with Smad4, and the complex translocates to the nucleus where it is involved in regulating transcriptional responses on target genes [10]. Ultimately, the predominant effect of TGFβ is to promote ECM accumulation.

Astragalus (*Astragalus membranaceus*) has long been known in traditional Chinese medicine as an immune-modulating herb. In clinical practice, administration of astragalus has achieved widespread use in the treatment of diabetes, and in the treatment of kidney abnormalities caused by diabetes [11,12]. Polysaccharoses, astragaloside, isoflavones, and saponin glycosides are the primary astragalus extracts [13]. Recent studies have demonstrated that astragalus has an antifibrotic effect in a rat model, and can inhibit the expression of TGFβ1, reduce ECM synthesis, and block tubular epithelial-to-mesenchymal transition (EMT) processes [14,15]. Results of a meta-analysis revealed that astragalus injection had therapeutic effects in DN patients, including reduced urine protein and improved renal function [16]. Astragaloside IV, one of the main active ingredients of astragalus, was shown to ameliorate podocyte apoptosis, prevent acute kidney injury, and attenuate glycated albumin-induced EMT in renal proximal tubular cells [17,18].

Hence, understanding the mechanisms underlying the administration of astragalus as a treatment for DN is essential for its application in clinical therapy. To this end, we employed a DN model to investigate the effects of astragalus injection on the TGFβ/Smad pathway.

## Methods

### Chemicals and reagents

Astragalus injection was purchased from the Chengdu di’aojiuhong pharmaceutical factory, Chengdu, China.

### Experimental animals and treatment

All experiments were performed in accordance with the guidelines on Ethical Standards for investigations in animals, and the study was approved by the Beijing University of Chinese Medicine animal research committee. Sixteen male KKAy mice (9–11 weeks of age) weighing 25–28 g were used in the current experiments. Eight male C57BL/6J mice (9–11 weeks of age) weighing 23–25 g were used as age-matched controls. All mice were purchased from the Animal Center of the Chinese Academy of Medical Science (Beijing, China), and were raised in the Clinical Institute of China-Japan Friendship Hospital (Beijing, China). During the conduct of the experimental protocol, the KKAy mice were allowed access to high-fat diet (HFD) and water *ad libitum*. To serve as a control, the C57BL/6J mice were fed a normal diet and allowed *ad libitum* access to water.

At 14 weeks of age, blood samples were obtained from the tail vein of the mice for the purpose of measuring blood glucose. A mouse with blood glucose levels above 13.9 mM was considered diabetic. The KKAy mice were randomly divided into the model group (MG, n = 8), and the treatment group (TG, n = 8), with an equivalent distribution of average body weights and blood glucose levels between the two groups. The C57BL/6J mice were used as the control group (CG, n = 8). The treatment group received daily intraperitoneal injections of astragalus (0.03 ml/10 g.d), while the model group received an injection of an equivalent volume of saline. The mice were housed individually in plastic cages with *ad libitum* access to food and water throughout the experimental periods.

Weekly body weight measurements were conducted and no differences were detected among the groups. Blood samples for the determination of blood glucose levels were taken from the tip of the tail every four weeks using the BREEZE2 Blood Glucose Test Strips (Bayer HealthCare, USA). At 24 weeks of age, all the mice were deprived of food pellets for 10 h, and blood was collected from the orbital plexus. Samples were kept on ice for 1 h, and the plasma was separated by centrifugation at 2000 rpm for 15 min at 4°C. Plasma samples were subsequently stored at -20°C until analysis. A portion of the kidney tissues collected were excised and frozen instantly in liquid nitrogen for the polymerase chain reaction (PCR) and western blotting assays. The remaining tissue was fixed in 4% buffered paraformaldehyde for hematoxylin and eosin (HE) staining and Masson staining.

### Biochemical analysis

Mice were euthanized after the collection of blood samples at 24 weeks of age. Blood urea nitrogen (BUN) and plasma creatinine (CREA) levels were measured by an Automated Biochemical Analyzer (Hitachi, Japan).

### Urine albumin analysis

At 24 weeks, mice were transferred to metabolic cages for 24 h for the purpose of collecting urine samples. Urine albumin concentrations were determined using an automatic biochemistry analyzer (DADE Xpand, USA). Albuminuria in mice was expressed as milligrams (mg) per 24 h.

### Renal histological analysis

Kidney sections were fixed in 4% buffered paraformaldehyde, embedded in paraffin, and cut into 4-μm-thick sections which were prepared for HE and Masson staining.

### Analysis of TGFβ1, TGFβR-I, Smad3, and Smad7 mRNA expressions by Reverse transcriptase-PCR

Total RNA was extracted from the kidney samples using Trizol (Invitrogen, CA, USA), and the total RNA concentration and purity were determined by measuring the OD260 and OD280 ratio. RNA was reverse-transcribed using GoScript Reverse Transcription System (Promega, USA), following the manufacturer protocol. Primers for PCR (Table 1) were designed and synthesized by Sangon Biotech Co., Ltd (Shanghai, China). PCR reactions were performed in a thermal cycler (Bio-Rad Laboratory, USA) using the following conditions: 95°C for 5 min followed by 36 cycles of 95°C for 30 s, 50°C for 30 s, 72°C for 40 s, and 72°C for 8 min. The PCR products were analyzed by gel electrophoresis using a 1% agarose gel. The quantity of specific mRNA was normalized as a ratio to the amount of β-actin mRNA.

**Table 1:** PCR sequences and PCR products

**Name**	**Length**	**Upstream primer (5′-3′)**	**Downstream primer (5′-3′)**
TGF-β1	493 bp	TCCCTCAACCTCAAATTATTCA	GCGGTCCACCATTAGCAC
TGFβ-RI	172 bp	GGCGAAGGCATTACAGTGTT	TGCACATACAAATGGCCTGT
Smad7	309 bp	ACAGAAAGTGCGGAGCAAGAT	CTGATGAACTGGCGGGTGTAG
Smad3	232 bp	GGGCCAACAAGTCAACAAGT	CTGGCTGGCTAAGGAGTGAC
β-actin	243 bp	GAAATCGTGCGTGACATTAAGG	CACGTCACACTTCATGATGGAG

### Western blot analysis for TGFβ1, TGFβR-I, Smad3/7 and p-Smad3

The lysates were clarified by centrifugation and the supernatants collected. Protein concentrations were determined using the bicinchoninic acid assay (BCA) Protein Assay (Applygen, Beijing, China). Equivalent amounts of tissue protein (80 μg) were resolved on SDS polyacrylamide gels, and transferred by electroblotting to polyvinylidene difluoride (PVDF) membranes. The membranes were blocked in 5% (W/V) nonfat milk at room temperature for 1 h, and then incubated overnight at 4°C with the primary antibody against Smad3 (dilution 1: 200, Santa Cruz, CA, USA), Smad7 (dilution 1:200, Santa Cruz, CA, USA), p-Smad3 (dilution 1:1000, Epitomics, CA, USA), and β-actin (dilution 1: 1000, Santa Cruz, CA, USA). After washing in a buffer containing Tris-buffered saline (TBS), with 0.1% Tween, the membranes were incubated with horseradish peroxidase (HRP)-linked anti-mouse secondary antibody at a dilution of 1:3000. Following washing in 0.1% Tween TBS buffer, the immunolabeled proteins were detected by enhanced chemiluminescense reagents (Applygen, Beijing, China). The density of the detected bands was analyzed by Quantity One software.

### Statistical analysis

Numerical data were expressed as means ± standard deviation (SD) of at least three independent experiments. Differences in group means were examined using analysis of variance (ANOVA), and resulting *P* values < 0.05 were considered statistically significant.

## Results

### Astragalus injection controls blood glucose levels and body weights

No apparent fluctuations in behavior or physiological appearance were noted among mice in the control group. However, the mice in the model group exhibited depression, reduced activity, and a lackluster coat, all of which are typical manifestations of diabetes. Despite exhibitions of symptoms similar to those of the model group, the clinical indications exhibited by the treatment group were milder.

**Figure 1:**
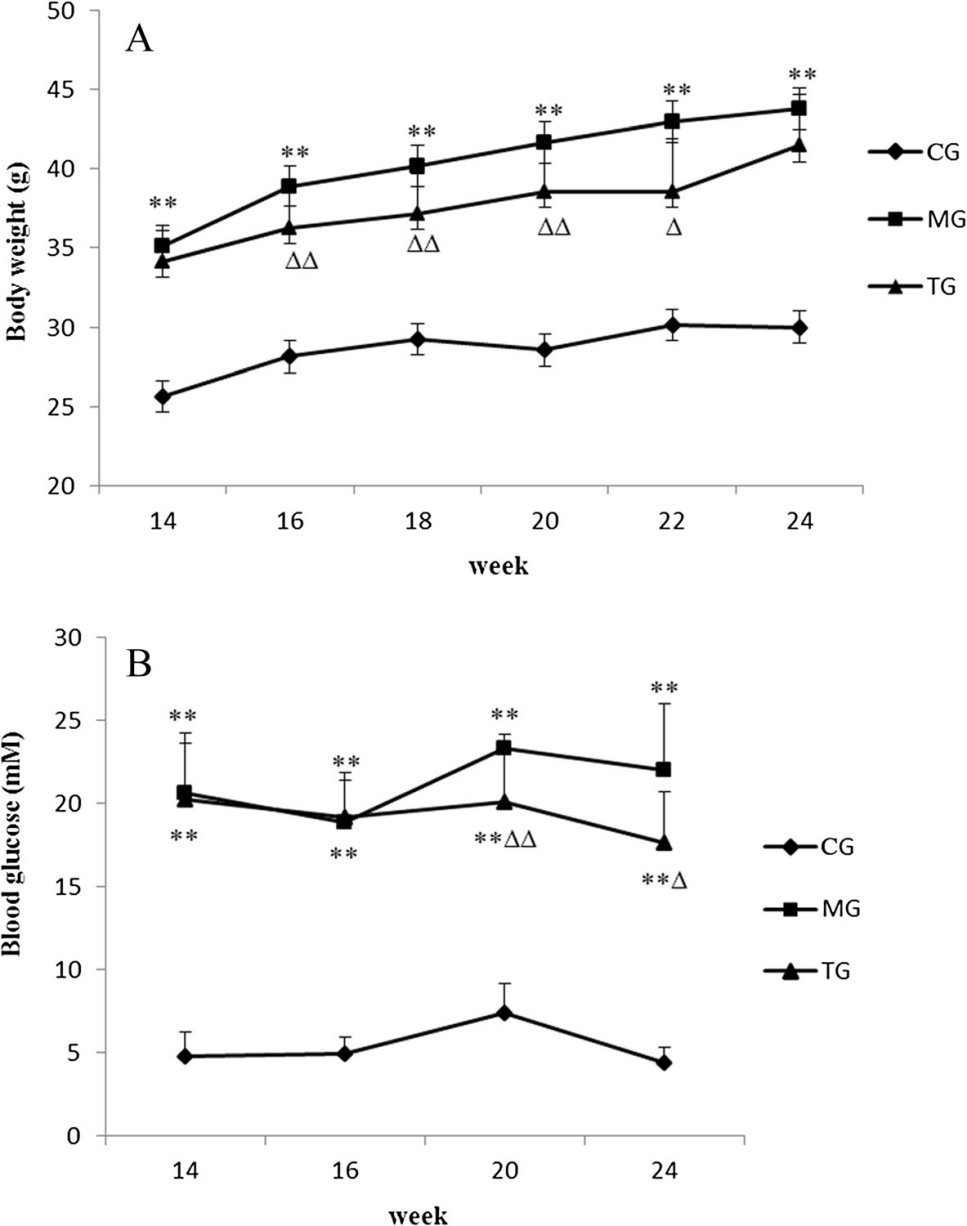
**Body weights and blood glucose levels at different weeks.** The bar graphs summarize average values for body weight and blood glucose. Body weights gradually increased in the model group mice, which indicated that astragalus treatment could significantly inhibit a gain in body weight **(A)**. Diabetic mice (MG) remained hyperglycemic and C57BL/6J mice (CG) remained normoglycemic throughout the period of study. The tendency for astragalus treatment to reduce the blood glucose concentration in diabetic mice was apparent. However, blood glucose levels did not return to normal following the administration of astragalus **(B)**. Data presented are means ± SD (n = 6–8). CG = the control group, MG = the model group, and TG = the astragalus treatment group. Compared with NG, *P < 0.05, **P < 0.01. Compared with MG, ^Δ^*P* < 0.05, ^ΔΔ^*P* < 0.01.

The body weights of the mice in the model group were significantly higher (*P* < 0.01) than body weights in the control group (Figure 1A), which persisted for the duration of the study. Though the body weights of mice in the treatment group also increased gradually, the mean weight of treatment group mice was significantly lower than weights of mice in the model group, as evidenced by measurements taken at 16,18, 20, and 24 weeks, respectively (Figure 1A). The blood glucose levels in the model group were also increased (*P* < 0.01) compared with blood glucose levels in the control group (Figure 1B). Elevated blood glucose levels were significantly reduced (*P* < 0.01); however, after astragalus treatment at 20 and 24 weeks (Figure 1B). Nonetheless, the treatment with astragalus was not able to reduce blood glucose levels to within normal ranges.

### Astragalus injection reduced albuminuria and deterioration of renal function

**Figure 2:**
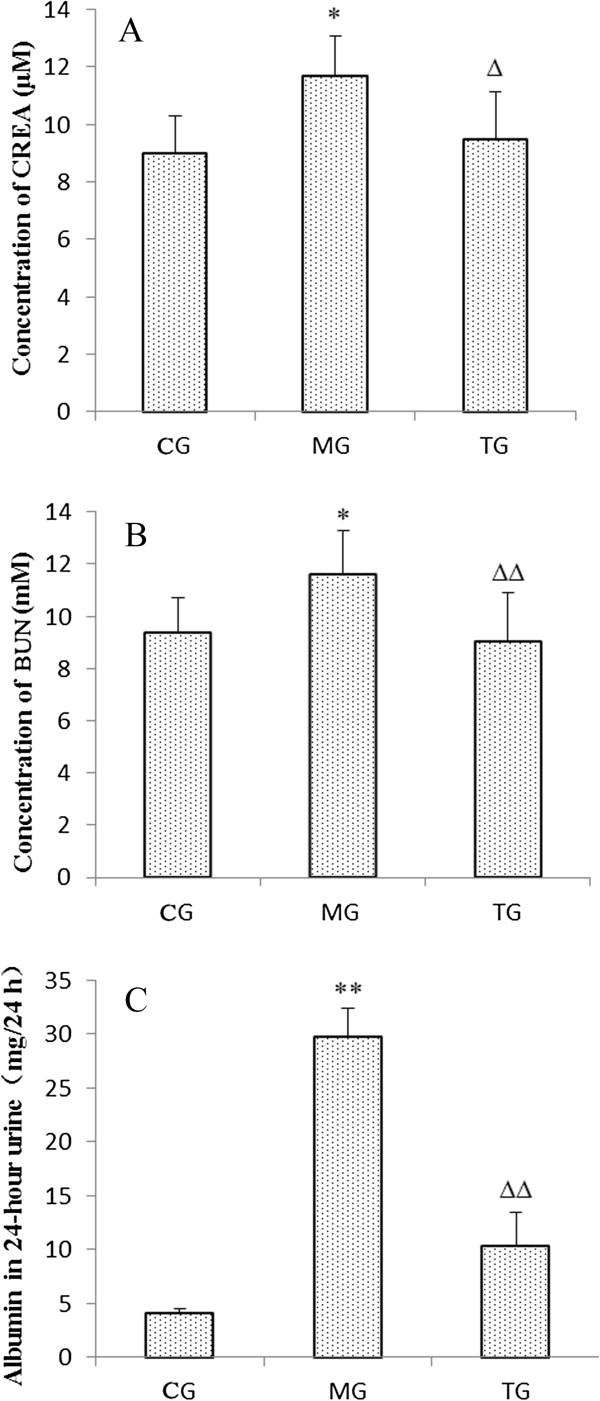
**The concentration of plasma creatinine, blood urea nitrogen, and urine albumin at 24 weeks of age.** Mean plasma creatinine was elevated in the MG, while increases in plasma creatinine with diabetes was prevented in the TG **(A)**. Blood urea nitrogen levels were similar to plasma creatinine, and increased in the MG but decreased in the TG **(B)**. Urine albumin also increased in the MG, which was markedly reduced with astragalus treatment **(C)**. Data presented are means ± SD (n = 6–8). CG = the control group, MG = the model group, and TG = the astragalus group. Compared with NG, *P < 0.05, **P < 0.01. Compared with MG, ^Δ^P < 0.05, ^ΔΔ^*P* < 0.01.

At 24 weeks of age, significant differences (*P* < 0.01) were found in plasma concentrations of creatinine, blood urea nitrogen, and albumin between the control group and the model group, (Figure 2). The treatment group showed significantly lower levels of plasma creatinine (*P* < 0.05), blood urea nitrogen *(P* < 0.01), and urine albumin (*P* < 0.01) following administration of astragalus when compared to the model group (Figure 2).

### Astragalus injection prevented morphological changes in the kidneys of diabetic mice

**Figure 3:**
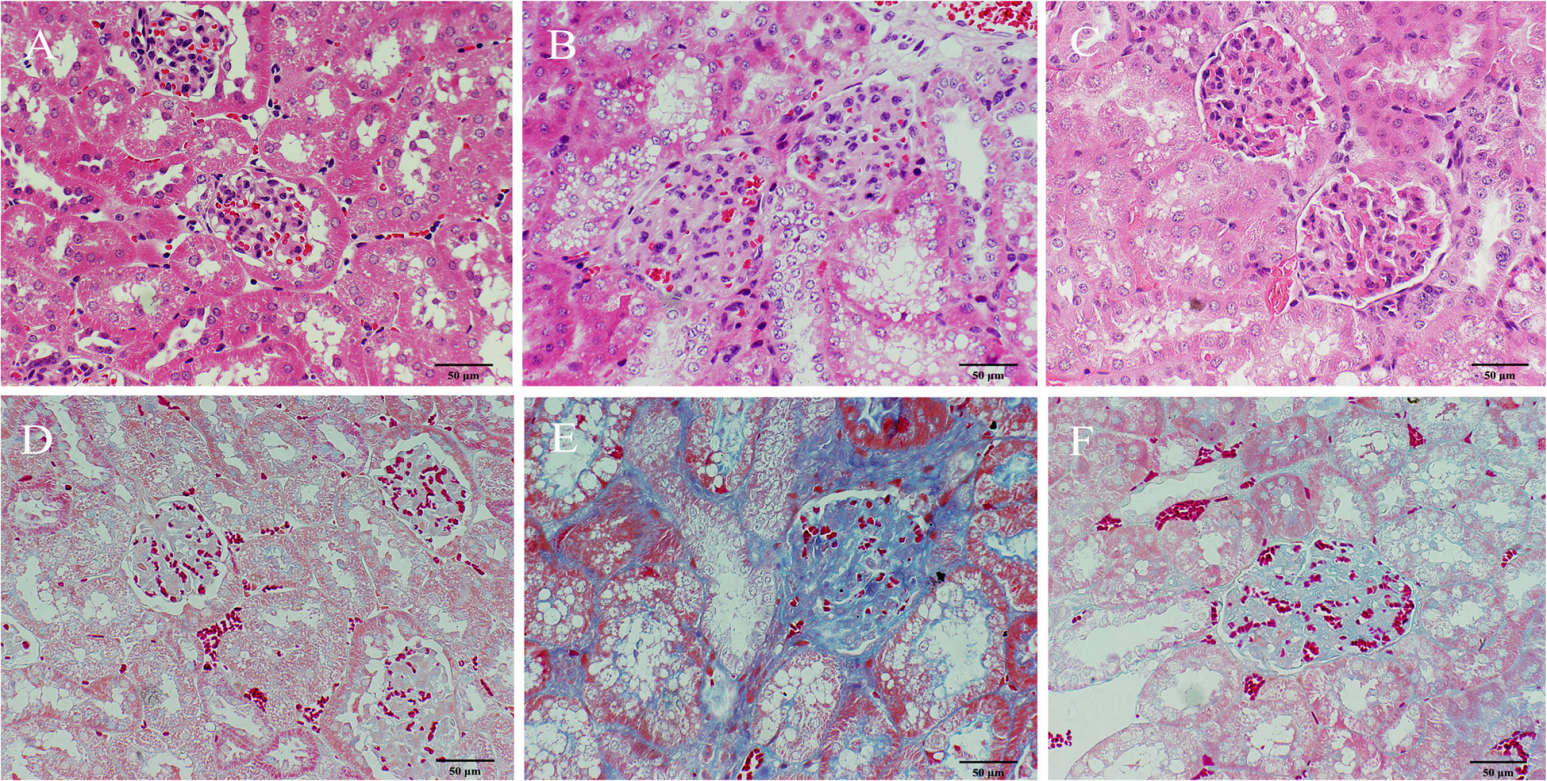
**Renal pathology of the different groups.** Pathological damage in KKAy mice at 24 weeks of age. Glomerulosclerosis and interstitial fibrosis are the main pathology in diabetic nephropathy. **A-C**: HE staining, 400X. The CG **(A)**, the MG **(B)**, and the TG **(C)**. HE staining revealed a thickened basal membrane and vacuolar degeneration in the renal tubular epithelial cells. D-F: Masson staining, 400X. The CG **(D)**, the MG **(E)**, and the TG **(F)**. Masson staining revealed collagen deposition (blue color) in the interstitium and glomeruli, which indicated improved renal pathology.

To identify pathological damage in the kidney, and to confirm the protective effect of astragalus in DN, kidney sections were processed for HE and Masson staining. Compared to the control group, a variety of DN-induced changes in renal morphology were detected in pathology results from the model group, including thickening of the basal membrane and vacuolar degeneration in the renal tubular epithelial cells (Figure 3A-C). Masson staining revealed obvious glomerular sclerosis and interstitial fibrosis in KKAy mice. However, treatment with astragalus reversed these changes to some degree (Figure 3).

### Effects of astragalus injection on the expression of Smad3, Smad7, TGFβ1, and TGFβR-I at the mRNA level

**Figure 4:**
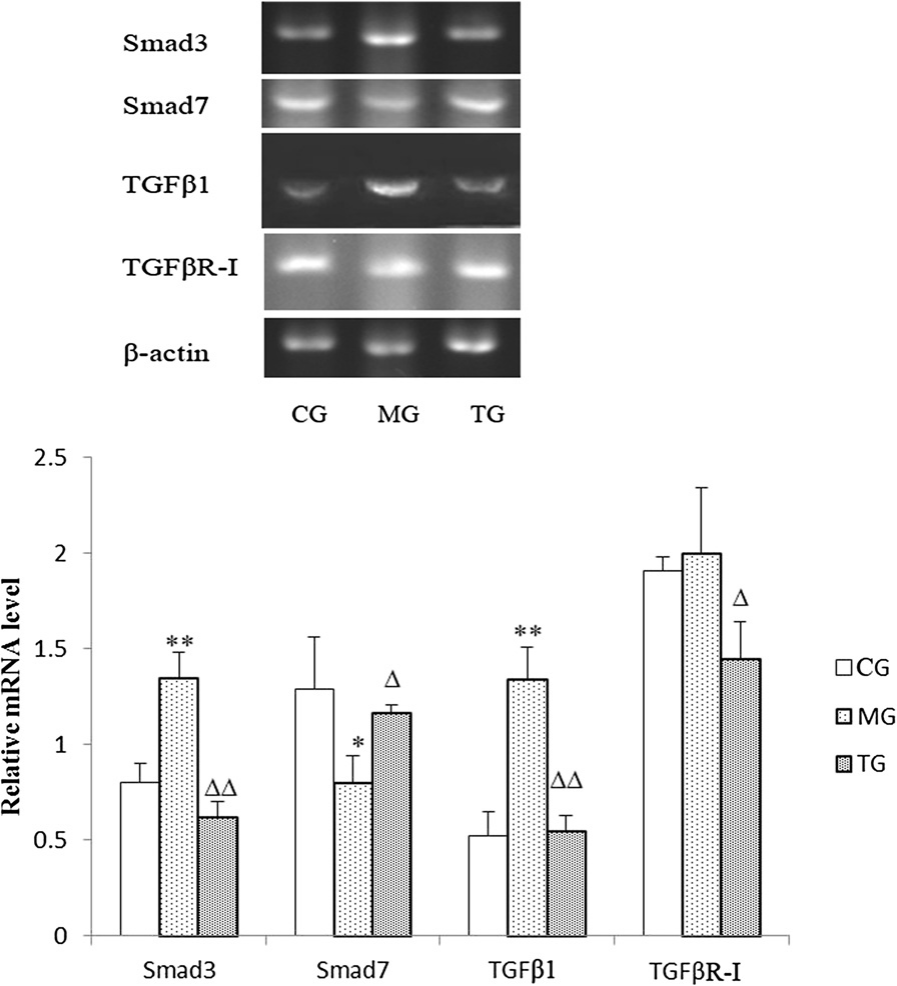
**Astragalus injection had effects on the mRNA expression of Smad3, Smad7, TGFβ1, and TGFβR-I.** Mice were sacrificed at week 24. The MG showed decreased expression of Smad7 and increased expression of Smad3, TGFβR-I, TGFβ1. Data presented are means ± SD (n = 3). CG = the control group, MG = the model group, and TG = the astragalus group. Compared with CG, *P < 0.05, **P < 0.01. Compared with MG, ^Δ^*P* < 0.05, ^ΔΔ^*P* < 0.01.

Using reverse transcriptase-PCR, we found that administration of astragalus injection significantly modulated the mRNA expression of Smad3, Smad7, TGFβ1, and TGFβR-I in kidneys of diabetic mice. Significant reductions in the relative amounts of Smad3 (*P* < 0.01), TGFβ1 (*P* < 0.01), and TGFβR-I (*P* < 0.05) mRNA were apparent in diabetic mice treated with astragalus compared to relative levels in the model group (Figure 4). Conversely, expression of Smad7 was significantly lower (*P* < 0.05) in KKAy mice when compared to expression in the normal mice (Figure 4). Further, treatment with astragalus injection increased Smad7 expression markedly (*P* < 0.05) in diabetic mice (Figure 4).

### Effects of astragalus injection on the expression of Smad3, Smad7, TGFβ1, TGFβR-I, and p-Smad3 at the protein level

**Figure 5:**
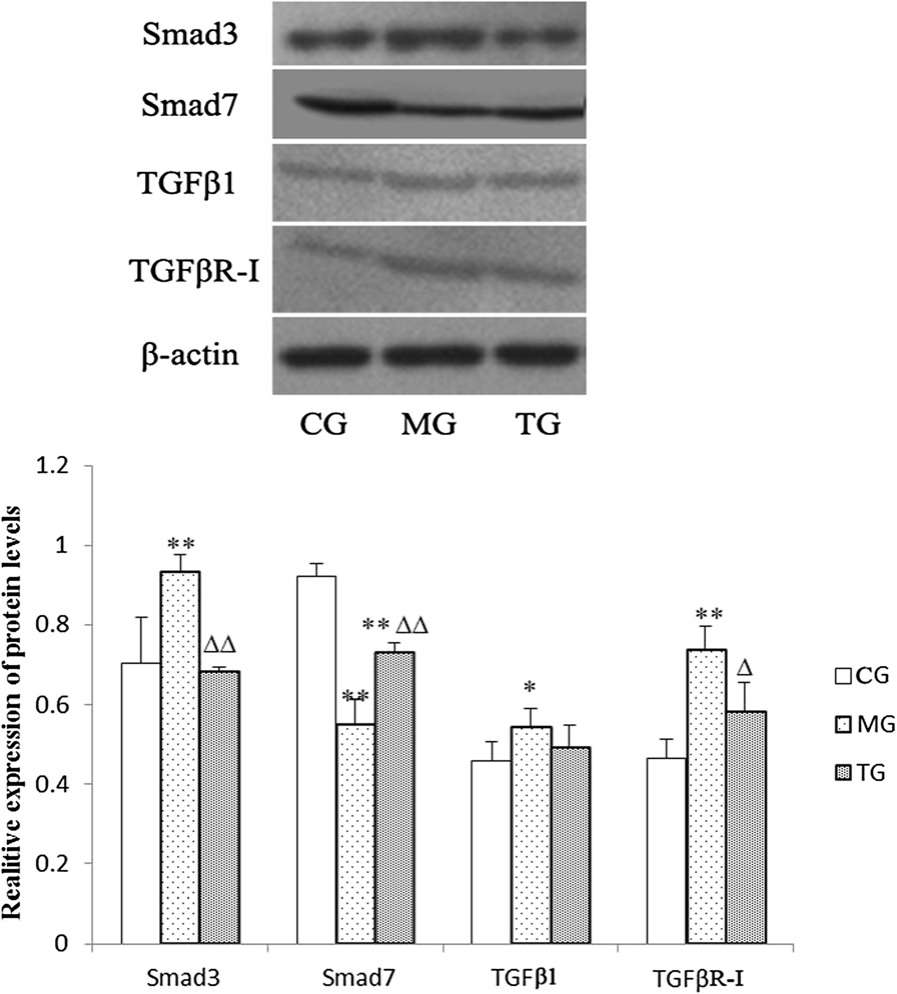
**Astragalus injection had effects on the protein expression of Smad3, Smad7, TGFβ1, and TGFβR-I.** Protein extracts from the renal cortex were analyzed to evaluate the expression of TGFβ/Smad proteins. The reduction in Smad3 and TGFβR-I protein levels was similarly observed in diabetic mice after astragalus treatment, but no significant change was observed in the TGFβ1 level. CG = the control group, MG = the model group, and TG = the astragalus group. Compared with CG, *P < 0.05, **P < 0.01. Compared with MG, ^Δ^*P* < 0.05*,*^ΔΔ^*P* < 0.01.

Western blot analysis was used to examine the expression of Smad3, Smad7, TGFβ1, and TGFβR-I. The model group displayed significantly higher levels of Smad3 (*P* < 0.01), TGFβ1 (*P* < 0.05), and TGFβR-I (*P* < 0.01), and notably lower levels of Smad7 (*P* < 0.01) when compared to levels measured in the control group (Figure 5). Treatment with astragalus significantly reversed increases in Smad3 (*P* < 0.01) and TGFβR-I (*P* < 0.05) proteins, as well as the apparent decrease in Smad7 (*P* < 0.01) protein in diabetic mice. However, treatment with astragalus had no effect on TGFβ1 expression (Figure 5).

**Figure 6:**
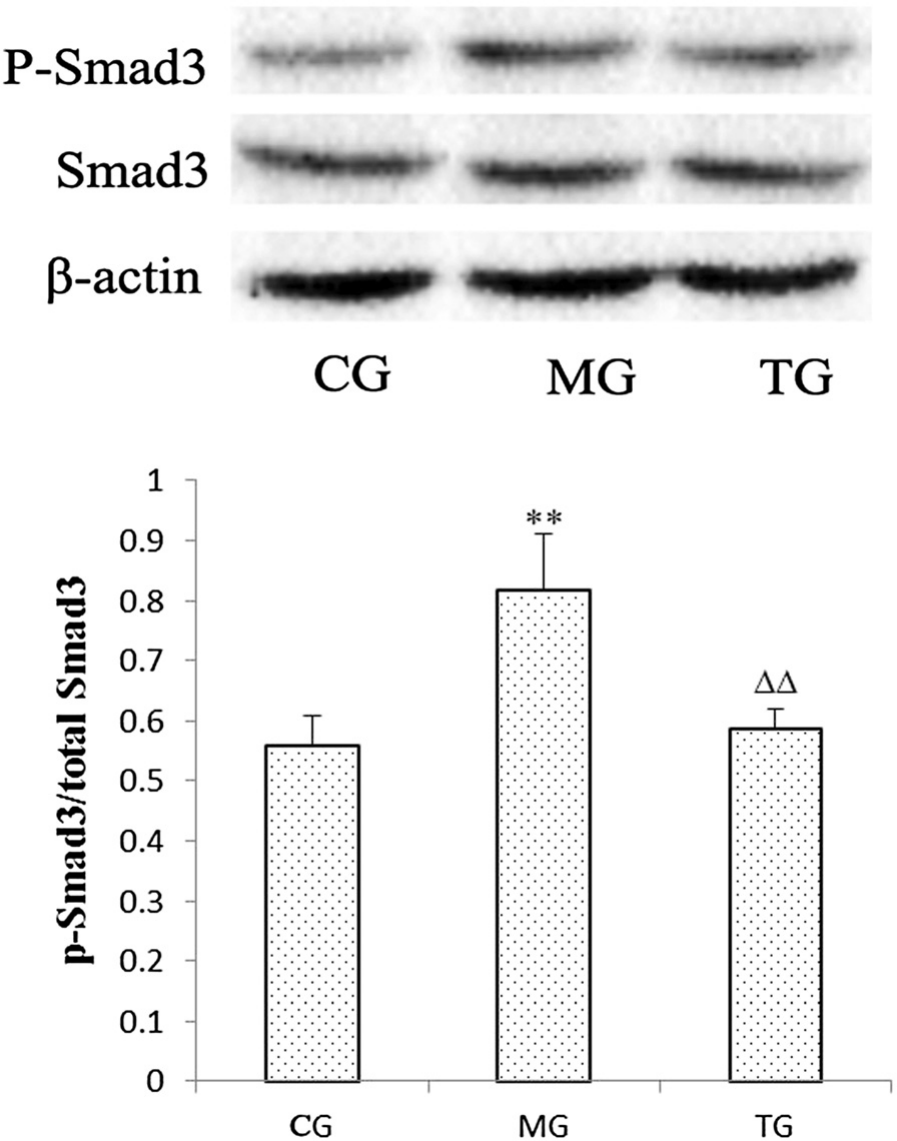
**Smad3 phosphorylation in diabetic mice was suppressed by astragalus injection.** Smad3 was actived in diabetes. The ratio of phosphorylated Smad3 to total Smad3 was increased in KKAy mice. In addition, Smad3 activation was significantly inhibited in diabetic mice treated with astragalus. Representative western blots of p-Smad3 and total Smad3 are shown. CG = the control group, MG = the model group, and TG = the astragalus group. Compared with CG, *P < 0.05, **P < 0.01. Compared with MG, ^Δ^*P* < 0.05*,*^ΔΔ^*P* < 0.01.

Because Smad3 is activated by phosphorylation, we examined the expression of phosphorylated Smad3 by western blot. The results demonstrated that the renal phosphorylation of Smad3, as a fraction of total Smad3, was significantly increased (*P* < 0.01) in the model group when compared with the control group (Figure 6). Conversely, treatment with astragalus significantly inhibited (*P* < 0.01) the activation of Smad3 (Figure 6).

## Discussion

Diabetic nephropathy, one of the most frequent chronic microvascular complications of diabetes mellitus, is the leading cause of end-stage kidney failure [19,20]. The main pathological changes manifest in DN are accumulation of ECM, degeneration of tubular epithelial cells, atrophy, and even the disappearance of some tubules, thickening of basal membranes, and infiltration of inflammatory cells in the mesenchyme [21,22]. The KKAy mouse, a well-established model of type-2 diabetes, was produced by transferring the yellow obese gene (Ay allele) into the KK/Ta mouse [23]. In a previous study, KKAy mice developed obesity, hyperglycemia, and albuminuria by 14-weeks of age. Furthermore, HE staining demonstrated thickening of the glomerular basement membrane, and vacuolar degeneration in the renal tubular epithelial cells. Further, Masson staining of histological sections revealed obvious glomerular sclerosis and interstitial fibrosis in the KKAy mouse, which was consistent with findings in previous studies [22].

The Chinese herb astragalus is an effective medical prescription used clinically for the treatment of DN [16,24]. All of the major constituents of astragalus have been shown to differentially lower high blood glucose levels and improve impaired glucose tolerance in models of type 2 diabetes [25,26]. In the current study, injection of astragalus for 10 weeks produced a mild hypoglycemic effect as suggested in our data above. However, glycemic control using insulin was demonstrated to ameliorate DN in STZ-DM rats [27], which suggested that astragalus administration produces other renoprotective mechanisms beyond sugar lowering effects. Diabetic albuminuria is an early hallmark of DN, and is always associated with the development of characteristic histopathology features. Results including aggravated kidney injuries such as albuminuria, glomerular sclerosis, and interstitial fibrosis in KKAy mice further supported this notion. However, treatment with astragalus attenuated theses changes in diabetic kidneys. The idea that injections of astragalus can improve renal function through inhibiting the EMT process and subsequent collagen production may provide further support of these results [28]. Moreover, it was found that levels of serum creatinine and blood urea nitrogen were significantly elevated in the KKAy mouse. Furthermore, the treatment group exhibited milder symptoms compared to the model group following the injection of astragalus, which indicated that astragalus administration could ameliorate the deterioration of renal function. Taken together, administration of astragalus may be appropriate for controlling blood glucose levels and body weight. Specifically, astragalus can reverse changes in renal histopathology and attenuate albuminuria, which could lead to improvement in renal function. Thus, there is a need to investigate the molecular mechanisms of astragalus administration for the treatment of DN.

We likewise aimed to investigate the mechanism of astragalus administration as a treatment for DN by focusing on the TGFβ/Smad pathway. TGFβ, a multifunctional cytokine that leads to renal fibrosis, plays a crucial role in the pathogenesis of DN. Interestingly, the administration of TGFβ neutralizing antibodies significantly reduced renal fibrosis [9]. In our previous studies, we concluded using immunohistochemistry that TGFβ1 protein levels were elevated in diabetic kidneys of the KKAy mice. Furthermore, we also found that TGFβ1 was mainly expressed in the cytoplasm of the renal tubular epithelial cells, and was rarely expressed in glomeruli [29]. In the current study, the amount of TGFβ1 expression was higher in the model group than in the normal group, which was in accord with previously reported findings [30,31]. Additionally, treatment with astragalus significantly reduced TGFβ1 mRNA expression, which suggested that astragalus may play a role in the down-regulation of TGFβ1 at the transcriptional level. Our western blot analyses confirmed that TGFβR-I proteins were up-regulated in the model group; however, increased TGFβR-I expression was not significant at mRNA levels. This phenomenon may be explained by the possibility that the mRNA of TGFβR-I is more prone to degradation, due to its unstable nature.

One of our aims in the current experiment was to investigate the effects of astragalus administration on Smad3 and Smad7. Smad7 is one of the inhibitory Smads, and is known to block the TGFβ signaling pathway by inhibiting Smad2/3 phosphorylation, and thus exerting its anti-fibrotic effect [32]. Increasing evidence has indicated that disruption of Smad7 may accelerate renal fibrosis [33,34]. In our studies, the KKAy mice that exhibited the down-regulation of Smad7 developed more severe renal dysfunction, which provided further confirmation of the effect of Smad7 disruption on renal fibrosis. In addition to the inhibitory Smads, there are receptor-regulated Smads that can transduce the TGFβ signal, such as Smad2 and Smad3. Smad3 is a critical downstream mediator responsible for renal fibrosis that has been shown to function in the diabetes-induced up-regulation of fibronectin and α3 (IV) collagen, and that may play a critical role in the early phase of DN [35,36]. It has been well documented that Smad3-deficient mice are protected from renal fibrosis by a reduction in EMT, collagen deposition, and the expression of profibrotic TGFβ target genes [37,38]. As indicated by our results, the expression of Smad3 and phosphorylated Smad3 (pSmad3) were increased in the kidneys of diabetic mice. Furthermore, the fact that the administration of astragalus suppressed the expression and phosphorylation of Smad3, and promoted the expression of Smad7, which resulted in improved renal conditions, provides further evidence for the effectiveness of astragalus in the treatment of DN.

## Conclusions

In conclusion, we believe that DN is caused by an imbalance in the TGFβ/Smad pathway, and that as a result, fibronectin (FN) increases and assembles, which in turn leads to glomerular sclerosis and interstitial fibrosis. Consistent with our theory, the astragalus injection appeared to alleviate DN by suppressing Smad3 and p-Smad3, as well as the expression of TGFβR-I. Concurrently, astragalus also exerts its effect by reducing the mRNA level of TGFβ1, and promoting Smad7 expression. Collectively, these results demonstrated that astragalus administration could be a potential treatment for DN, and that astragalus could ameliorate the outcomes associated with DN by hindering the TGFβ/Smad pathway.

## Abbreviations

TGFβ: Transforming growth factor-beta; DN: Diabetic nephropathy; TGFβR-Ι: Transforming growth factor-beta receptor Ι; BUN: Blood urea nitrogen; CREA: Plasma creatinine.

## Competing interests

The authors have declared that there is no conflict of interest.

## Authors’ contributions

YN carried out the animal experiments, performed the statistical analysis and drafted the manuscript. YY participated in the western blot assay. WS involved in the extraction of RNA and RT-PCR, XC and DJ participated in the HE staining and discussion of the experiment. SL and QW formulated the original ideas and working hypothesis. QW is the owner of the research grant and revised the draft of manuscript. All authors read and approved the final manuscript.

## Pre-publication history

The pre-publication history for this paper can be accessed here:

http://www.biomedcentral.com/1472-6882/14/148/prepub
